# Is guideline-driven prophylaxis for venous thromboembolism common practice in the South African private hospital setting?

**DOI:** 10.4102/safp.v62i1.5022

**Published:** 2020-10-12

**Authors:** Melissa van der Merwe, Marlene Julyan, Jesslee M. du Plessis

**Affiliations:** 1 Department of Medicine Usage in South Africa, Faculty of Health Sciences, North-West University, Potchefstroom, South Africa

**Keywords:** venous thromboembolism, prophylaxis, hospital, South Africa, risk assessment

## Abstract

**Background:**

Prophylactic venous thromboembolism (VTE) strategies have the greatest impact on patient outcomes. Both global and local guidelines support VTE prophylaxis for hospitalised patients. However, studies have reported that these measures are routinely under-prescribed. This study evaluated prescribing patterns of VTE prophylaxis in one of the largest South African (SA) private hospital groups.

**Methods:**

A quantitative, retrospective analysis of the hospital group’s patient database was conducted for patients admitted between 01 September 2015 and 31 August 2016. Those younger than 18 years with trauma or suffering from contraindications to anticoagulation were excluded. Additionally, patients with warfarin billed were also excluded as they possibly required therapeutic anticoagulation. Included prophylactic measures were compared with published SA guidelines by abstracting prophylaxis type and dosing, according to corresponding individual patients’ VTE risk ratings.

**Results:**

Amongst the 373 020 patients included as the study population, 77% required prophylaxis. Of these, 38.36% (*n* = 85 486) received guideline-appropriate prophylactic measures during their hospital stay. Patients in whom prophylaxis was indicated, only 24.56% (*n* = 42 715) complied with the SA guidelines. The most commonly used prophylactic measures were enoxaparin (89.09%) and fondaparinux (2.68%). Prophylactic measures differed per speciality, with the most compliant amongst intensivists. A low uptake of the risk assessment model use (*n* = 222 860, 59.75%) was, however, reported for this data set.

**Conclusion:**

Less than 24.56% of patients who required prophylaxis received guideline-appropriate interventions. Further studies should focus on understanding differences in practice and improving acceptance and application of guideline-driven care.

## Introduction

The global incidence of venous thromboembolism (VTE) has been reported to be exceptionally high with an annual overall prevalence rate similar to that of stroke, the fifth leading cause of death worldwide.^1^ A multinational, observational, cross-sectional survey was carried out with 1583 patients from five sub-Saharan countries. A 50.4% VTE prevalence rate was reported amongst at-risk patients from urban specialised hospitals. The majority (62.3%) of those at risk were classified under a medical speciality, whilst surgical patients made up 43.8% of sufferers.^2^ For South Africa, the risk of VTE development during 2008 in hospitalised patients from parts of its Gauteng province was described to be at 74.6%, with venous thromboembolic-related deaths reported at around 20 000 persons annually in 2012.^3,4^ A lack of updated data currently exists, and because most VTE symptoms remain undetected, the true VTE incidence together with its possible impact on the South African (SA) private healthcare system largely remains unknown.^5,6^

Venous thromboembolic disease not only is debilitating but also presents a high economic burden on a country’s healthcare system. This is mainly because of a 45% increased cost for recurrent hospitalisation with VTE-related comorbid diseases.^7^ Recurring VTE-related hospitalisations often require a 48% increased expenditure when compared with the initial admission period.^8^ It is estimated that the highest cost is suffered during the first 3 days after re-hospitalisation, possibly because of the higher level of care required.^9^ Up to 24% of patients diagnosed with VTE will eventually require intensive care unit re-admission.^9^ In 2014, VTE-related hospitalisation cost the United States healthcare system around $10 billion, and in 2017, it was established through personal communication that one of South Africa’s largest private hospital groups spent over R195 million on VTE prophylactic and treatment measures.^7^ This may indicate VTE management being one of the most expensive medical strategies.

Venous thromboembolic development has traditionally been attributed to patients already hospitalised for extended periods of time.^10^ However, approximately 25% – 40% of non-hospitalised patients are at risk of VTE development.^10^ Pharmacological VTE prophylaxis is unfortunately not without hazard, as its underlying mechanism may result in life-threatening haemorrhage.^11^ It is therefore important to avoid pharmaceutical prophylaxis when the benefit does not outweigh the risk.^12^ For these reasons, patient risk stratification on admission is paramount in order to ensure that at-risk patients receive the correct type and dose of prophylaxis without incurring additional harm.^12^ It has been proven that supplementary to risk stratification, implementation of VTE prophylactic guidelines results in improved patient outcomes.^13^ Yet, VTE-related complications because of poor prophylactic practices have given rise to 64.4% of all premature deaths in high-income countries and 66% in low- and middle-income countries.^14,15^ This may be attributed to variances in prophylaxis used on patients.^15,16,17,18^ The SA arm of ‘The Use of VTE prophylaxis in relatioN to patiEnt risk profiling’ (TUNE-IN) study found that only 67.9% of patients rated as possessing a high risk for VTE development received appropriate prophylaxis.^3^ Interestingly, it is reported that the main reason for reduced prophylactic prescribing is prescribers’ perception that patients have a decreased risk for VTE development compared to available epidemiological data.^3^ A large discrepancy often exists in the perceived VTE development risk amongst patients clinically appraised versus those in whom a standardised risk assessment model (RAM) was completed.^3^ Risk assessment models are traditionally designed to select patients in whom VTE prophylaxis benefit would outweigh the risk.^3^ Several reviews exist on the different available RAMs’ ability to predict VTE development; however, no consensus has been reached on a preferred model.^19,20^ The American College of Chest Physicians’ (ACCP) VTE prophylaxis guidelines are generally regarded as the gold standard to be followed, and Caprini devised a RAM to enable the easy implementation of these guidelines.^13,21^ The Caprini RAM is founded on a point-based scoring method where points are awarded to risk factors according to their propensity for VTE.^13^ Based on the total calculated per patient, a low, medium or high risk for VTE development is awarded.^13^ The RAM by Caprini is also the only model that has been externally validated for its VTE prediction ability and can be used in medical as well as surgically ill patients.^22,23,24^ A modest reduction was furthermore detected in VTE prevalence after patients were screened using the Caprini RAM and its suggested prophylaxis initiated.^22^ This RAM possibly positions itself firmly as a tool to reduce VTE occurrence in patients, improve patient care and reduce medical-related expenses when used correctly.

Venous thromboembolism is considered one of the most expensive and common preventable causes of global mortality. Despite its implication, there exists a scantiness of data available to describe VTE risk and prophylactic measures across the private hospital sector in South Africa (which is required to reduce healthcare costs). Local, peer-reviewed VTE prophylactic guidelines have been published by the South African Society of Thrombosis and Haemostasis (SASTH).^25^ These guidelines are based on those prescribed by the ACCP, are peer reviewed and are user-friendly in their application. This study aimed at describing the VTE risk and prophylactic practices in the private sector across a large area of South Africa, by comparing practices to those prescribed by SASTH guidelines. This may possibly lay the foundation for patient outcome improvement.

## Methods

### Study design

A quantitative, retrospective data analysis was performed. The sample included all patient data of those admitted over a 1-year period as ‘inpatients’. Patients admitted as day cases or outpatients were excluded from the analysis. Data were analysed by the Department of Statistics, Faculty of Health Sciences at North-West University in South Africa.

### Setting

A private hospital group was selected as the study setting, as prescriber habits are not governed by healthcare sector formularies or restrictions. The study setting comprised 54 hospitals located in seven of the nine SA provinces (no representation for Limpopo and the Northern Cape and only one hospital for the North West province). Services rendered included general medicine, surgery (acute or specialised), obstetrics and gynaecology, internal medicine, cardiology and medical oncology as part of a tertiary hospital offering. Most admissions (23.98%, *n* = 106 085) for the study period were for referrals and admissions under general practitioners, with 22.48% (*n* = 94 455) admissions for surgically related procedures and 14.22% (*n* = 62 914) admissions for gynaecological or obstetric procedures.

### Data source

**FIGURE 1:**
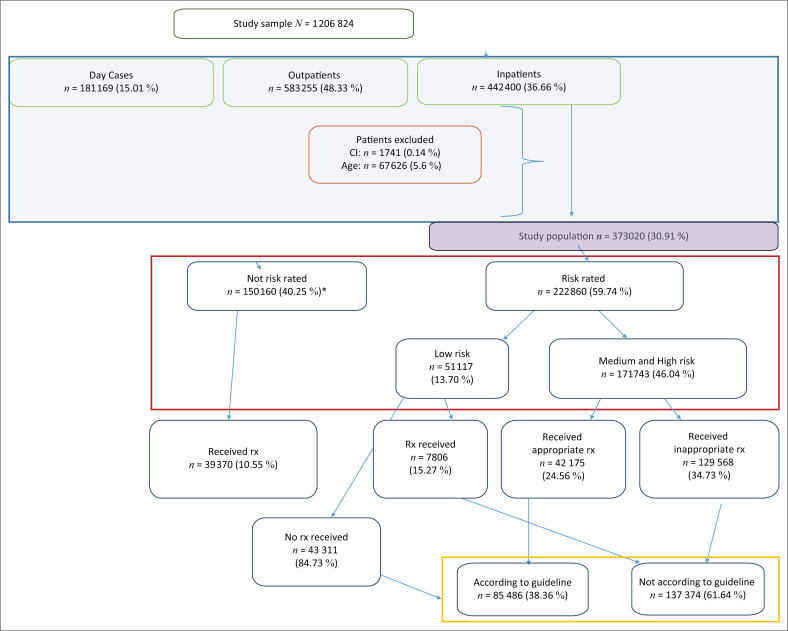
Data for study population.

All admission and coding information, according to the 10th revision of the International Statistical Classification of Diseases and Related Health Problems (ICD10), could be cross-linked to in-hospital pharmacy billing data. This enabled the abstraction of the type of prophylactic use with its dosing, frequency as well as duration. Prophylactic medicine data could then be cross-linked to the principal diagnosis and the VTE risk of the individual patient could be calculated by using unique patient admission numbers. Venous thromboembolism risk ratings were calculated by nursing staff during patient admission, as is the standard practice for the group. This was performed by utilising a modified version of the Caprini RAM with results captured by clinical case managers on patient admission profiles. Venous thromboembolism risk ratings were captured in 59.74% (*n* = 222 860) of the study participant data meeting the inclusion criteria. Risk ratings were absent for 40.25% (*n* = 150 160) of the patient data. As a result of the nature of the study, patients without VTE risk ratings’ data had to be excluded from guideline comparison, as no inference could be made on the appropriateness of prophylactic use. A summary of each risk factor and score, as contained in the modified Caprini RAM, is presented in Table 1 and a diagrammatical representation of the study sample data handling is outlined in Figure 1.

**TABLE 1:** Risk assessment model from study setting.

Modified Caprini RAM risk category	Patient characteristics
Low VTE development risk (risk factors assigned 1 point each)	Patients between 41 and 60 years of age Body mass index of > 25 Patients currently suffering from swollen legs Varicose veins Medical patient currently at bed rest Planned minor surgery Myocardial infarction (acute) Abnormal pulmonary function/chronic obstructive pulmonary disease History of inflammatory bowel disease History of prior major surgery in the last 30 days Suffering from congestive heart failure in the last 30 days Sepsis in the last 30 days Different lung diseases including pneumonia in the last 30 days Women who are pregnant or postpartum 30 days Women who are taking oral contraceptives or hormone replacement therapy Females with a history of unexplained stillborn babies or having had more than three recurrent spontaneous abortions, toxaemia resulting in premature births or patients with an infant showing slowed growth
Medium VTE development risk (risk factors assigned 2 points each)	Age between 61 and 74 years Those with a central venous line Current or prior malignancy Immobilised patients with plaster cast in the last 30 days Patients undergoing arthroscopy Immobilised patients of 72 h and longer Planned surgery of longer than 44 min
High VTE development risk (risk factors assigned 3 points each)	Patients older than 75 years Patient history of deep vein thrombosis and pulmonary embolism Patient familial thrombosis history
High DVT development risk (risk factors assigned 5 points each)	Those suffering multiple trauma in the last 30 days Patients suffering from paralysis or acute spinal cord injuries during the last 30 days Those with pelvic or hip fractures during the last 30 days Patients with planned hip or knee orthoplastic replacement

DVT, deep vein thrombosis; RAM, risk assessment model; VTE, venous thromboembolism.

The modified Caprini RAM differed from the originator by classifying obesity as having a body mass index (BMI) of 25, whereas Caprini’s classification included a BMI of over 30. Further differences included the exclusion of creatinine clearance, factor V Leiden levels, prothrombin 20210A levels, serum homocysteine levels, anticardiolipin antibody as well as Lupus antibody tests in the modified RAM. The modified Caprini RAM remains useful and was verified for settings where it may be impractical to conduct tests such as Lupus antibody assays because of time or financial constraints.^26^

### Data collection

Data consisted of all consecutive inpatient admissions from 01 September 2015 to 31 August 2016. Only data of those admitted as ‘inpatients’ were included, as these patients would be at greatest risk of reduced mobility. Patient data were excluded for those whose anticoagulant use was contraindicated or not described by SASTH guidelines. These included patients younger than 18 years, those with hepatic failure and any haemorrhagic linked condition, those admitted with traumatic brain injury or patients receiving therapeutic anticoagulation (*n* = 69 367; Figure 1).

**FIGURE 2:**
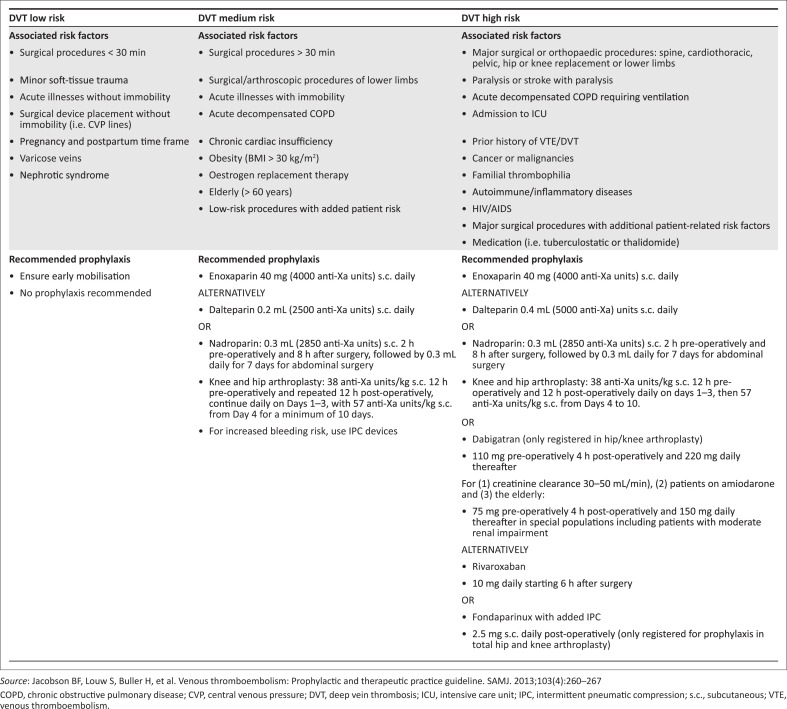
South African Society of Thrombosis and Haemostasis prophylactic practice guidelines for venous thromboembolism.

All included patient billing data were compared to the ‘Venous Thromboembolism: Prophylactic and Therapeutic Practice Guideline’ of the SASTH (Figure 2), and compliancy to this guideline was captured.^25^

### Statistical analysis

The Statistical Analysis System^®^, SAS 9.3^®^ (SAS Institute Inc., USA), was used to analyse the data. Categorical variables were reported as frequencies and percentages. For this proposed study, the Pearson’s chi-square test was used to determine the association between SASTH guideline compliance and clinical speciality. Cohen’s *d* value was used to determine the practical significance of the results (with *d* ≥ 0.1 defined as an effect with practical significance).^27^

### Ethical consideration

The Health Research Ethics Committee (HREC) of the Faculty of Health Sciences of the North-West University granted ethical approval, ethics number: NWU-00080-17-A1. Written permission was obtained from the study hospital group’s Ethics in Research Committee.

## Results

There were 373 020 inpatient records that met the inclusion/exclusion criteria for the study period. The mean age of the study population was 49.08 years (SD, 17.96 years) with a 38% to 62% male-to-female split. Patients were grouped together if they had undergone risk rating measures and further divided between those who would not require prophylaxis (low VTE risk) and those who would (medium or high VTE risk). Risk ratings were not available for 40.25% (*n* = 150 160) of the study population and contrasts with SASTH guidelines as the individualisation of prophylaxis according to VTE risk rating is recommended. Only 38.36% (*n* = 85 486) of all risk-rated patients in this data set received guideline-appropriate VTE prophylactic measures.

Of those risk rated, 13.70% (*n* = 51 117) were reported as having a low VTE development risk. Although guidelines do not recommend chemoprophylaxis for such patients, a total of 15.27% (*n* = 7806) did receive some form of medicinal intervention. For those rated as medium-to-high VTE risk (prophylaxis indicated), only 24.56% (*n* = 42 175) received guideline approved prophylaxis. This may point to a disconnect between VTE rating outcomes versus clinician diagnosis.

A data summary for patients who received prophylaxis, regardless of being risk rated, was added (Table 2) in an attempt to compare characteristics for patients in whom clinicians deemed prophylaxis as appropriate. Type of prophylaxis prescribed remained consistent amongst these patients. Significantly younger patients (41.10 years) were rated as low risk compared to those at moderate to higher risk. The top three ICD10 admission codes for those rated as low DVT risk (when compared to the SASTH guidelines), however, place these ‘low-risk’ patients at either a medium or a high risk for VTE development. The accuracy of VTE risk ratings performed by admitting nursing staff remains in question.

**TABLE 2:** Patient characteristics in those receiving prophylaxis.

Patient demographics	Not rated (*n* = 39 370)	Low risk rated (*n* = 7806)	Medium and high risk rated (*n* = 56 347)
Median age in years (IQR)	54.22 (SD = 17.94)	41.10 (SD = 14.38)	54.9 (SD = 17.8)
Gender – Female	60.80% (*n* = 23 931)	67.40% (*n* = 5261)	61.30% (*n* = 34 558)
Gender – Male	39.20% (*n* = 15 439)	32.60% (*n* = 2544)	38.70% (*n* = 21 788)
Gender – Unknown	0.00% (*n* = 0)	0.00% (*n* = 1)	0.00% (*n* = 1)
Primary admission	Maternal care due to uterine scar, 4.30% (*n* = 1685)	Maternal care due to uterine scar, 5.7% (*n* = 444)	Maternal care due to uterine scar, 4.40% (*n* = 2490)
	Unstable angina, 3.8% (*n* = 1483)	Pneumonia, unspecified, 2.7% (*n* = 210)	Unstable angina, 3.00% (*n* = 1714)
	Pneumonia, unspecified, 2.10% (*n* = 809)	Unstable angina, 2.2% (*n* = 175)	Congestive heart failure, 2.90% (*n* = 1632)
**Prophylaxis billed**			
Anticoagulant received	Enoxaparin, 91.80% (*n* = 36 139 doses)	Enoxaparin, 86.2% (*n* = 19 176 doses)	Enoxaparin, 89.90% (*n* = 33 447 doses)
	Fondaparinux, 2.6% (*n* = 1014 doses)	Fondaparinux, 2.7% (*n* = 1759 doses)	Fondaparinux, 2.60% (*n* = 1703 doses)
	Rivaroxaban 10 mg, 1.2% (*n* = 457 doses)	Rivaroxaban 10 mg, 1.6% (*n* = 997 doses)	Heparin, 3.99% (*n* = 2513 doses)
**Clinician specialty**			
Primary specialty	General practitioner, 22.90% (*n* = 9023)	Gynaecologist and obstetrician, 28.60% (*n* = 2235)	Gynaecologist and obstetrician, 24.26% (*n* = 13 668)
	Gynaecologist and obstetrician, 17.10% (*n* = 6740)	General practitioner, 22.8% (*n* = 1781)	Physician, 16.67% (*n* = 9395)
	Orthopaedic surgeon, 12.40% (*n* = 4871)	Physician, 13.10% (*n* = 1026)	General practitioner, 13.05% (*n* = 7355)

IQR, interquartile range; SD, standard deviation.

Characteristics for patients who received guideline-appropriate prophylaxis are set out in Table 3. Admission codes were found to be highest for ‘maternal care because of uterine scar from previous surgery’ (5.01%), and ‘unspecified viral hepatitis without hepatic coma’ (1.49%) was found to be the second most prevalent. Risk ratings in this subset of patient data favoured that of a high risk for VTE development (82.90%). Results revealed that high-risk-rated patients most often received enoxaparin or fondaparinux as prophylaxis.

**TABLE 3:** Characteristics of patients who received South African Society of Thrombosis and Haemostasis compliant prophylaxis.

Population prophylaxis, those compliant to SASH	*n*	Frequency (%)
**Primary ICD10**		
Maternal care due to uterine scar from previous surgery	2113	5.01
Unspecified viral hepatitis without hepatic coma	629	1.49
Unstable angina	629	1.49
Other primary gonarthrosis	507	1.20
Spinal stenosis, lumbar region	488	1.16
Delivery by elective caesarean section	438	1.04
Adult hypertrophic pyloric stenosis	437	1.04
Adult respiratory distress syndrome	437	1.04
Stroke, not specified as haemorrhage	403	0.96
Bronchitis, not specified as acute or chronic	402	0.95
Bronchopneumonia, unspecified	402	0.95
Other and unspecified intestinal obstruction	393	0.93
Sepsis, unspecified	390	0.92
Insulin-dependent diabetes mellitus	368	0.87
Primary gonarthrosis, bilateral	351	0.83
Chronic obstructive pulmonary disease with acute exacerbation	350	0.83
Chronic obstructive pulmonary disease, unspecified	350	0.83
Gluteal tendinitis, pelvic region and thigh	325	0.77
Gonarthrosis, unspecified	325	0.77
Excessive and frequent menstruation with irregular cycle	302	0.72
Chronic kidney disease, stage 5	284	0.67
Malignant neoplasm of parotid gland	276	0.65
Malignant neoplasm of prostate	276	0.65
Acute renal failure, unspecified	268	0.64
Acute respiratory failure, Type 1 (hypoxemia)	268	0.64
Liver disorders in pregnancy, childbirth	262	0.62
Lobar pneumonia, unspecified	262	0.62
Lumbago with sciatica, site unspecified	256	0.61
Lumbar and other intervertebral disc disease	256	0.61
**Risk**		
DVT HIGH	34 820	82.56
DVT MEDIUM	7355	17.44
**Gender**		
Female	26 942	63.88144635
Male	15 233	36.11855365
**Mean age**		
56.45	-	18.19
**Prophylaxis**		
Enoxaparin	-	87.24
Fondaparinux	-	4.04
Heparin	-	3.59
Rivaroxaban (10 mg)	-	2.67
Fraxiparine	-	1.20
Nadroparin	-	0.27
Dabigatran	-	0.27
Mechanical prophylaxis	-	0.72

Note: *n* = 42 175, 24.56%.

SASTH, South African Society of Thrombosis and Haemostasis.

It was found that enoxaparin took the highest share of prescribing chemoprophylactics by comparing the different molecules used as chemoprophylactic preventives (Table 2). Dosages of 40 mg subcutaneous every 24 h were mostly billed. Fondaparinux was the second highest prescribed agent at 2.5 mg subcutaneous every 24 h. Unfractionated heparin (UFH; which had a higher use in those compliant with the SASTH guidelines; Table 3) was used at a dosage of 5000 IU, every 8–12 h. A dose of 10 mg rivaroxaban novel oral anticoagulant (NOAC) was more commonly used in orthopaedic surgery patients; however, this was not limited to its registered indication for hip and knee arthroplasty. Prophylaxis used in surgery disciplines was highest for enoxaparin followed by dalteparin (NOAC). Prescribing of prophylaxis in medically ill patients favoured enoxaparin as a once in 24-h administration, followed by dalteparin at 5000 IU subcutaneous every 24 h. The recommended doses of low-molecular-weight heparin (LMWH), UFH and rivaroxaban appear to have been adhered to.

Whilst chemoprophylaxis formed the largest part of all types of prophylaxis used, mechanical prevention methods were utilised in 1.2% of patients (regardless of SASTH guideline-compliancy measure). Graduated compression stockings were slightly more favoured (0.66%) as the mechanical prevention method compared to the use of pneumatic compression sleeves (0.19%).

The compliance of prophylactic prescribing by speciality varied amongst prescribers (*d* > 0.1 and 0.000 < *p* > 0.003; Table 4). Intensivists (*d* = 0.42, *p* < 0.003), paediatricians and paediatric surgeons (*d* = 0.2, *p* < 0.000) generally were more likely to prescribe SASTH guideline-directed VTE prophylaxis (Table 4).

**TABLE 4:** Distribution of South African Society of Thrombosis and Haemostasis compliancy by doctor speciality.

Speciality	SASTH category	*n*	Observed probability	Exact Sigma (two tailed)	Cohen’s *d*
Cardiologist	Compliant	1963	0.27	0.000	0.23
	Non-compliant	5345	0.73	-	-
	Total	7308	1.00	-	-
General practitioner	Non-compliant	25 651	0.70	0.000	0.20
	Compliant	10 776	0.30	-	-
	Total	36 427	1.00	-	-
Gynaecologist and obstetrician	Non-compliant	16 776	0.63	0.000	0.13
	Compliant	9695	0.37	-	-
	Total	26 471	1.00	-	-
Intensivist	Non-compliant	1	0.08	0.003	0.42
	Compliant	12	0.92	-	-
	Total	13	1.00	-	-
Orthopaedic surgeon	Non-compliant	10 013	0.65	0.000	0.15
	Compliant	5282	0.35	-	-
	Total	15 295	1.00	-	-
Paediatric surgeon	Non-compliant	55	0.34	0.000	0.16
	Compliant	106	0.66	-	-
	Total	161	1.00	-	-
Paediatrician	Compliant	4	0.10	0.000	0.40
	Non-compliant	35	0.90	-	-
	Total	39	1.00	-	-
Physician	Non-compliant	11 260	0.66	0.000	0.16
	Compliant	5809	0.34	-	-
	Total	17 069	1.00	-	-
Surgeon	Non-compliant	12 983	0.70	0.000	0.20
	Compliant	5464	0.30	-	-
	Total	18 447	1.00	-	-
Urologist	Non-compliant	5751	0.83	0.000	0.33
	Compliant	1172	0.17	-	-
	Total	6923	1.00	-	-

SASTH, South African Society of Thrombosis and Haemostasis.

## Discussion

In this single-year retrospective study, less than a quarter of patients requiring VTE prevention (those rated as medium or high VTE risk) received guideline-appropriate intervention. This contrasts with the high VTE development risk found in over 77% of patients.

Mention should also be made of the reduced percentage of RAM application found in this study. Although a risk of over-classification of VTE prophylactic requirement in low-risk patients occurs with the use of a RAM, it has been proven to increase prescriber awareness of patients’ VTE risk.^3^ This is because it has been found that prescribers do not routinely risk assess patients and base prophylactic prescribing on their perceived patient VTE development risk.^3^ The routine use of RAMs, on the other hand, further prevents the under-diagnoses of patients at high risk of VTE in 20% of patients, as is recommended for use in the private sector of South Africa.^3^

The study population’s mean age was found to be less than that reported in the TUNE-IN study but close to the mean age of 45.15 years as with the ENDORSE-Africa study.^3,2^ The fact that this study population contained high gynaecological admission volumes as compared to other studies may explain the age differences. Female gender distribution was found to be similar to the SA arm of the TUNE-IN Wave 2 and ENDORSE-Africa (65.6%) studies.^2,20^ This reported percentage of at-risk patients is higher than the 74% average found in the TUNE-In Wave 2 study but within the range of 44% – 80% for the ENDORSE study.^17,20^ The rate of appropriate prophylaxis found is much lower than the rates from both the SA TUNE-IN Wave 2 (70.9%) and ENDORSE studies but falls in line with the ENDORSE-Africa study of guideline-compliance rates between 22% and 80%.^2,17,20^ A possible reason for the lower compliance in this study is that the study population for medically ill patients included all patients older than 18 years and risk rated as exhibiting a low, medium or high thrombosis risk. The ENDORSE methodology (as followed in ENDORSE-Africa and TUNE-IN Wave 2 studies), however, only included patients older than 40 years.^2,17,20^ The SASTH guidelines do not specifically mention age as a predictor of risk but advocates RAMs to be used to determine VTE risk.^25^ This resulted in adding RAM results and ICD10 coding as inclusion criteria. Upon comparing patient age amongst those requiring prophylaxis, findings revealed that older patients were more likely to have received prophylaxis compared with those who did not receive any form of prophylactic anticoagulation. A possible explanation for this finding is that 1.4% of patients, contained in the grouping which did not receive preventative measures, was admitted with ‘spontaneous vertex delivery’. These patients theoretically are assumed to be of a younger age and here the use of prenatal anticoagulation is off-label. The SASTH guidelines do recommend LMWH use prenatally in those where the benefit outweighs the risk; however, this practice requires much caution.^25^

This study further found that in gynaecological surgery (such as in the case of the study population’s highest admission portion), the use of enoxaparin 40 mg subcutaneous daily, starting between 6 and 8 h post-procedure, was prescribed. This is in compliance with the SASTH guidelines. The SASTH guidelines suggest the use of dalteparin for patients undergoing high-risk surgical procedures and is also registered as prophylaxis in patients at moderate risk of thrombosis (those undergoing abdominal and gynaecological surgery), as well as those presenting with a high risk of thrombosis (those undergoing orthopaedic surgery or suffering from an underlying malignancy). Study results reveal dalteparin use to be the second highest chemoprophylaxis molecule used in surgical procedures, which is in line with the guidelines. The high overall use of enoxaparin (89.90%) is very similar to the TUNE-IN Wave 2 study (92%) and is in line with SASTH recommendations where LMWH is stipulated as being superior to UFH for most prophylactic indications.^25,28^ One possible reason for the low use of mechanical prophylaxis in our current study population may be because of its recommended use in those at high risk of bleeding as well as the lower percentage of surgery-related admissions (excluding gynaecological surgery). Mechanical prophylaxis (pneumatic stockings) was found to be used in less than 3% of patients in the TUNE-IN Wave 2 study which is, however, higher than our study (1.2%).^28^

Whilst practices amongst specialities differed from published guidelines, no comment can be made on the appropriateness of the prophylaxis used. This is because no clinical outcome data were available or included in the study for patients at risk of thrombosis development. One possible reason for the variance in prescriber prophylaxis may be because of the fact that the last review of the SASTH guidelines occurred in 2013 and newer anticoagulants have since been registered in South Africa. Another factor may be patients’ bleeding risk (which may not have been ICD10 coded), as well as the risk of clinician litigation because of patient haemorrhage. The accuracy of VTE risk ratings may also play a role in prescribers deviating from the guidelines. It must be noted that this study’s aim was not to critique prescribing practices but to illustrate occurring prophylaxis patterns as well as patient VTE risk rating spread.

Results for this study reveal that younger, female gynaecology patients make out the bulk of those who are at risk of VTE development. With such patient demographics existing in the private sector and the fact that clinicians are relying more on their own diagnosis than the results achieved from RAMs, a possibility exists that private hospital patients may require more prophylaxis than that traditionally prescribed.

## Limitations of this study

The study data set is similar to the data utilised by medical aids to determine prescribers and hospital disease risk profiling. As this was a retrospective study dependent on claims information with linked pharmacy billing data, a potential exists for ICD10 case ascertainment and capturing errors, as well as under-coding. Nevertheless, because of the availability of pharmacy data, a more detailed data set was available, which enabled prescription-detail inclusion. A combination of inaccurate billing and VTE risk assessments may have contributed to the smaller percentage of patients found to have received prophylaxis in accordance with the SASTH guidelines. Primary ICD10 coding was used to select the study population – this may have inadvertently led to certain patients in whom anticoagulation was contraindicated, being included in the study.

## Conclusion

This study confirms that most prescriber specialities do not prescribe VTE prophylaxis according to published local, consensus-derived guidelines. It can further be concluded that patients are more likely to receive some form of VTE prophylaxis if they are at high risk of VTE development. This is similar to other study findings where patients at medium risk of VTE development did not receive the required VTE prophylaxis as stipulated in the guidelines. Differences in prophylactic patterns were found between clinician specialities; however, the clinical implication of this effect remains unknown. The application of RAMs was found to have been used in less than half of the admitted patients, possibly pointing to the fact that prophylactic prescribing is more reliant on clinician diagnosis than on risk assessment. This is in contrast to the guidelines suggesting that all admitted patients should be subjected to a RAM, together with a clinician review. The concern of bleeding risk in patients, and the costs associated with VTE prophylactic under-prescribing, should not be disregarded, therefore pointing to the importance of RAM outcomes to ensure only those at risk of thrombosis are treated. Possible future research to establish reasons for clinicians not utilising RAM rating outcomes in prescribing decisions may pave the way for an improved RAM utilisation, which, in turn, may standardise VTE prophylaxis to improve outcomes.

Further review of VTE prophylaxis guidelines to include newly registered anticoagulants, as well as methods to improve the completion and acceptance of RAMs may balance the current under- and overprescribing amongst the different VTE risk categories.
